# Multi‐omics and case‐control analyses identify immunoglobulin M as a tumour‐derived serum biomarker of ocular adnexal extranodal marginal zone lymphoma

**DOI:** 10.1002/ctm2.1259

**Published:** 2023-05-03

**Authors:** Jiahao Shi, Min Zhou, Xiaowen Zhou, Shichong Jia, Zhen Liu, Yi Zhao, Jiahui Shi, Xin Song, Yefei Wang, Renbing Jia, Yixiong Zhou, Xianqun Fan

**Affiliations:** ^1^ Department of Ophthalmology Shanghai Jiao Tong University School of Medicine Affiliated Ninth People's Hospital Shanghai P. R. China; ^2^ Shanghai Key Laboratory of Orbital Diseases and Ocular Oncology Shanghai P. R. China; ^3^ Tianjin Key Lab of Ophthalmology and Visual Science Tianjin Eye Hospital Tianjin P. R. China; ^4^ CAS Key Laboratory of Computational Biology Shanghai Institute of Nutrition and Health University of Chinese Academy of Sciences Chinese Academy of Sciences Shanghai P. R. China; ^5^ Department of Ophthalmology South Hospital of the Sixth People's Hospital Shanghai P. R. China

**Keywords:** biomarker, immunoglobulin mu, lymphoma, ocular adnexal lymphoma, proteomics

Dear Editor,

With integrative molecular profiling and case‐control profiling, we identified immunoglobulin M (IgM) as a tumour‐derived serum biomarker for ocular adnexal extranodal marginal zone lymphoma (OA‐EMZL) and further constructed a preoperative diagnostic model.

OA‐EMZL is the commonest subtype of ocular adnexal lymphomas (OALs),^1^ and the second common subtype of extranodal marginal zone lymphomas (EMZLs).^2^ It has similar clinical features and distinct treatment strategies compared with other orbital space‐occupying lesions.^3^ Few tools are designed for preoperative differential diagnosis of OA‐EMZL.^3^ A definite diagnosis of OA‐EMZL requires a biopsy and a complicated pathological examination. Together, these make the efficient differential diagnosis of OA‐EMZL an issue in clinical practice. Thus, we aim to find a biomarker for OA‐EMZL to facilitate preoperative decision‐making.

Herein, we investigated serum proteome and tissue transcriptome in 83 samples to identify potential tumour‐derived diagnostic serum biomarkers (Figure 1A–C and Tables S1 and S2).^4^, ^5^, ^6^, ^7^ Differentially expressed proteins (DEPs) detected by serum proteome are not identically secreted by tumour tissues. Based on previous research,^4^ we hypothesized that tumour‐derived biomarkers should be positively correlated in abundance between omics (Figure 1D), and concordantly differentially expressed in both omics (Figure 1E and Table S3). Immunoglobulin Heavy Constant Mu (IGHM) was the highest correlated gene (*R* = 0.56, *p* < 0.001), and the most dysregulated DEP in the proteome (Log2(foldchange) = 2.64, *p* < 0.001) (Figure 1D,E). The dysregulation of IGHM was consistent across subgroups in both proteome and transcriptome (Figure 1G).

**FIGURE 1:**
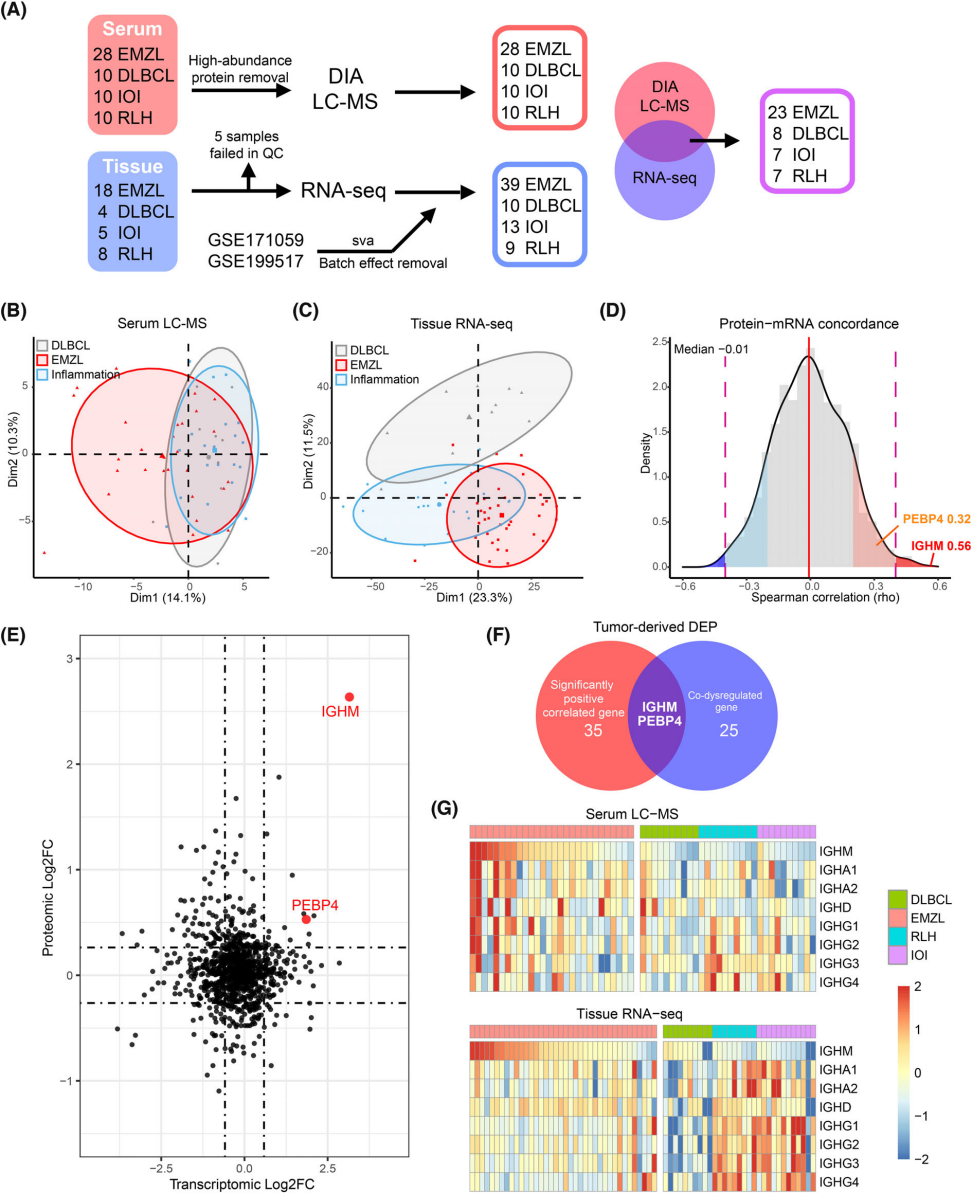
Proteotranscriptomic analysis identifies immunoglobulin M (IgM) as a tumour‐derived serum differentially expressed protein (DEP) of extranodal marginal zone lymphoma (EMZL). (A) Sampling workflow of tissue transcriptomic and serum proteomic cohort. (B) Principal component analysis (PCA) of high variant proteins (protein with top 25% median absolute deviation). (C) PCA of high variant transcripts (transcripts with top 25% median absolute deviation). (D) Density plot shows the across‐subject Spearman correlation for 1003 protein‐mRNA pairs. (E) Scatterplot shows differentially expressed genes (DEGs) of protein‐mRNA pairs identified in the transcriptomic cohort (38 EMZLs and 31 controls) and the proteomic cohort (28 EMZLs and 30 controls) between EMZLs and controls. The horizontal line is at proteomic |log2(FC)| = log2(1.2); vertical line is at transcriptomic |log2(FC)| = log2(1.5). (F) Venn plot of significantly positive correlated genes and co‐dysregulated genes identifies two tumour‐derived DEPs. (G) Heatmap of immunoglobulin constant region analyzed by proteomic and transcriptomic data. The dysregulation of Immunoglobulin Heavy Constant Mu (IGHM) is consistent across subgroups in tissue transcriptome and serum proteome. No similar dysregulated expression is found in other immunoglobulin constant regions.

IGHM encodes an indispensable constant region of the IgM heavy chain. EMZL originates from marginal zone B‐cell,^8^ which is the main producer of secreted IgM.^9^ Immunofluorescent staining suggested IgM was expressed in neoplastic cells instead of bystanders (Figure S1). The malignant B cells exhibit monotypic immunoglobulin expression with light chain restriction. We developed an “absolute κ/λ score” to quantify monoclonality in multi‐omics data. The score calculated by transcriptomic and proteomic data were both significantly higher in EMZLs compared with nonmalignant diseases (Figure S2A,B) and significantly correlated in EMZLs (Figure S2C). The serum score was highly correlated to the serum IGHM in EMZLs instead of controls (Figure S2D,E). This evidence supports the tumour‐derived nature of serum IgM and further attributes the elevated IgM to the neoplastic cells.

We investigated 205 patients with pathologically confirmed diagnoses who underwent serum immunoglobulin examination in a case‐control analysis (Table S4). Patients with OAL apart from EMZL, lymphoid hyperplasia and chronic orbital inflammation (idiopathic orbital inflammation, IgG4‐related disease, Granuloma, Mikulicz disease, Sarcoidosis disease, Kimura disease, Sjögren syndrome and Amyloidosis) were enrolled as controls. Serum IgM was significantly higher in EMZL compared with other subgroups and diagnoses (Figure 2A and Figure S3A). Among immunoglobulins, IgM exhibited a strong diagnostic potential in differentiating EMZLs from controls (Figure 2B). It also consistently distinguished EMZLs from subgroups and diagnoses (Figure S3B). Multivariate logistic regression indicated that serum IgM (*p* < 0.001) was the independent predictor for EMZL (Table S5). Paired IgM concentrations were available in 70 patients at diagnosis and after treatment. OA‐EMZL patients exhibited a significantly decreased serum IgM after initial treatment (Figure 2C). There was no significant correlation between serum IgM and clinicopathological features of EMZL patients (Figure 2D,F). The results indicated the consistency of serum IgM in EMZLs.

**FIGURE 2:**
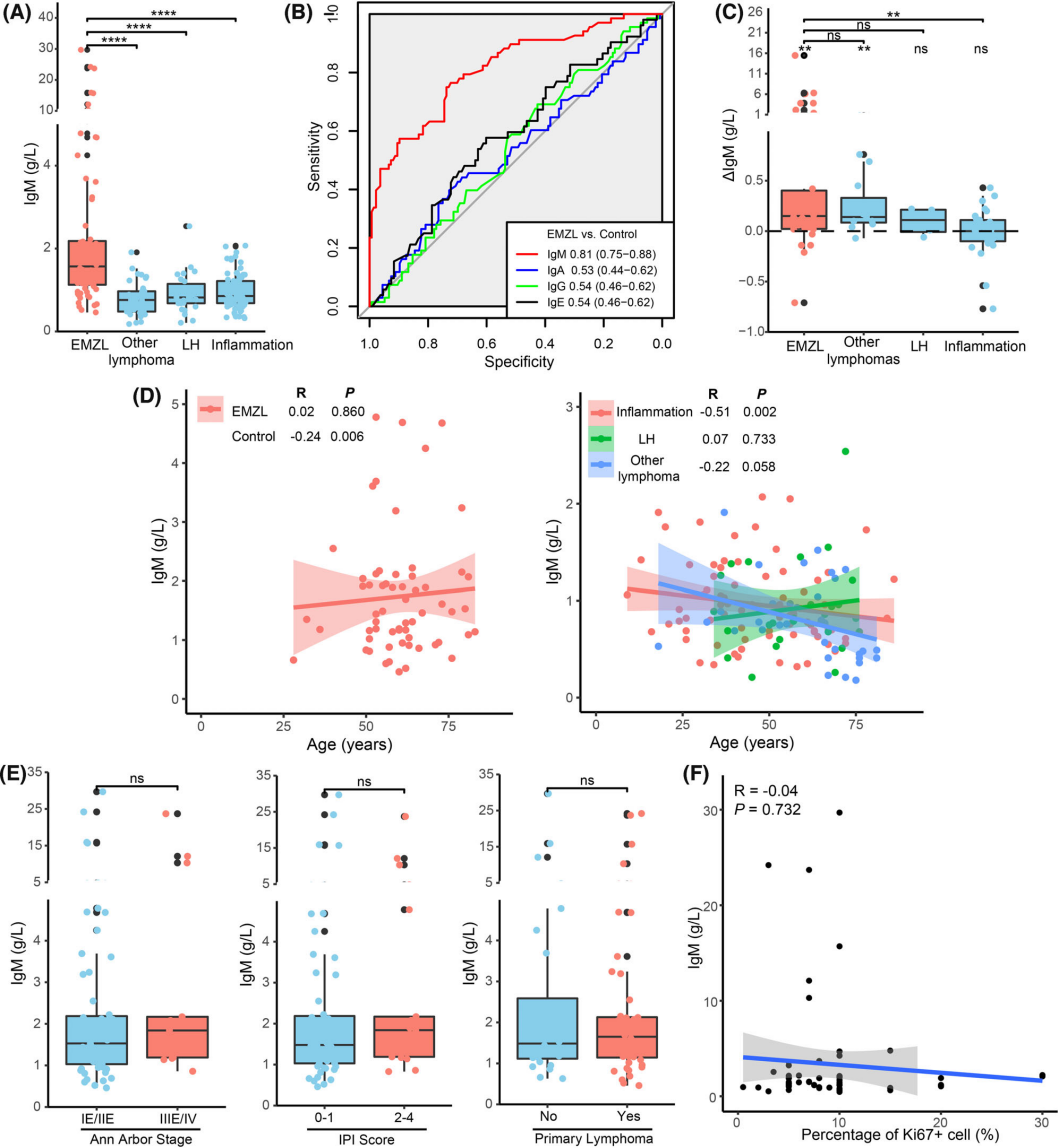
The clinical cohort demonstrates immunoglobulin M (IgM) as a tumour‐derived serum biomarker for extranodal marginal zone lymphoma (EMZL). (A) EMZL exhibits a significantly higher serum IgM concentration than other lymphoma subtypes, LH, and chronic inflammation. **** indicates *p* < 0.0001. (B) Receiver operating characteristic (ROC) plots of serum IgM, IgA, IgG, and IgE. IgM exhibited an area under the curve (AUC) value of 0.81 (0.75–0.88). Other immunoglobulins exhibited no diagnostic potential. (C) EMZL and other lymphoma subtypes exhibit a significantly higher ΔIgM (post‐treatment serum IgM concentration—pre‐treatment serum IgM concentration) compared with 0. EMZL exhibits a significantly higher ΔIgM compared with chronic inflammation. ** indicates *p* < 0.01; “ns” indicates no statistical significance. (D) Spearman's rank correlation shows serum IgM is significantly negatively correlated with age at diagnosis in the control group and chronic inflammation. It is not correlated with age in EMZL, LH, or other lymphoma subtypes. Lines indicate linear regression. (E) Serum IgM in EMZL is not associated with Ann Arbor stage, IPI score, or primary lymphoma. “ns” indicates no statistical significance. (F) Spearman's rank correlation shows serum IgM in EMZL is not correlated with Ki67.

Furthermore, we evaluated potential confounders based on the previous report.^10^ Different genders exhibited a similar serum IgM concentration in all subgroups (Figure S4A). Spearman correlation analysis showed a significant negative correlation between serum IgM and age in control and chronic inflammation groups (Figure 2D). Restricted cubic splines (RCS) analysis suggested serum IgM as a linear variable (Figure S4B), and age as a non‐linear variable (*p* for non‐linearity = 0.002) (Figure S5A). Thus, we defined an optimal cutoff for age at diagnosis as 47 years based on receiver operating characteristic (ROC) analysis and converted it into a category variable (Figure S5B).

To simplify the usage of serum IgM concentration in the clinical context, we conducted propensity score matching (PSM) to minimize the potential bias and further converted IgM into an ordinal variable (Figure 3A and Table S6). After PSM, serum IgM consistently distinguished EMZLs from controls (Figure 3B). IgM was fitted consistently as a linear variable (Figure 3C). The cutoff value of serum IgM concentration at 1.46 g/L (Specificity 0.897; Sensitivity 0.559) was determined by ROC; 0.88 g/L (Specificity 0.560; Sensitivity 0.853) was determined by the minimal Akaike information criterion (AIC) value of RCS (Table S7). The ordinal IgM exhibited a comparable area under the curve (AUC) compared with the continuous (Figure 3D,E).

**FIGURE 3:**
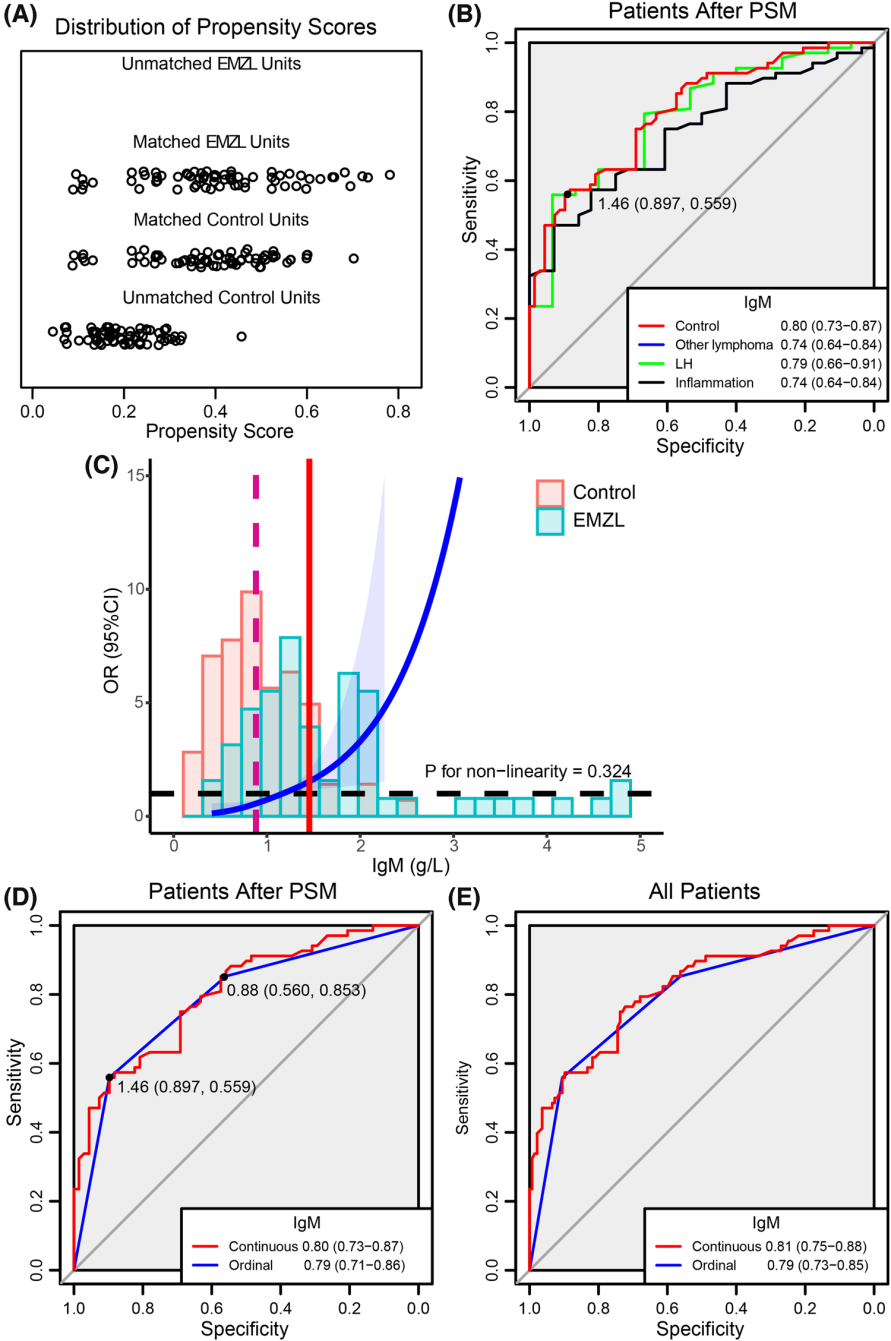
Propensity score matching (PSM) and restricted cubic splines (RCS) analyses identify the threshold of immunoglobulin M (IgM). (A) Scatter plot of the propensity score for patients. The propensity score for each individual is calculated given the covariates of sex, age, date of diagnosis, bilateral involvement, and disease site. 1:1 nearest neighbor matching is applied to ensure minimal bias. (B) Receiver operating characteristic (ROC) plot of serum IgM in patients after PSM. Serum IgM exhibits an area under the curve (AUC) significantly higher than 0.5 in distinguishing extranodal marginal zone lymphomas (EMZLs) from controls, other lymphoma subtypes, LHs, and chronic inflammations. (C) RCS analysis shows the relation of serum IgM with OR of EMZL in patients after PSM. Serum IgM is fitted consistently as a linear variable. The vertical lines represent the knots. (D) ROC plot of ordinal and continuous serum IgM in patients after PSM. (E) ROC plot of ordinal and continuous serum IgM in all patients.

Serum IgM solely is not sufficient for the preliminary diagnosis based on its AUC. Thus, we further constructed a diagnostic model. In the validation cohort, multivariate analysis showed IgM between 0.88–1.46 g/L (*p* = 0.017), IgM ≥1.46 g/L (*p* < 0.001), and age at diagnosis < 47 (*p* = 0.008) were independent predictors of EMZL (Table S8). Lacrimal gland involvement was included in the equation based on AIC. A nomogram entitled preliminary EMZL diagnostic model (preEM) was constructed based on the results (Figure 4A). Compared with continuous or ordinal IgM, preEM exhibited relatively higher discriminative ability in both derivation and validation sets (Figure 4B,C). The calibration plots exhibited a great agreement between nomogram predictions and actual observations (Figure 4D,E).

**FIGURE 4:**
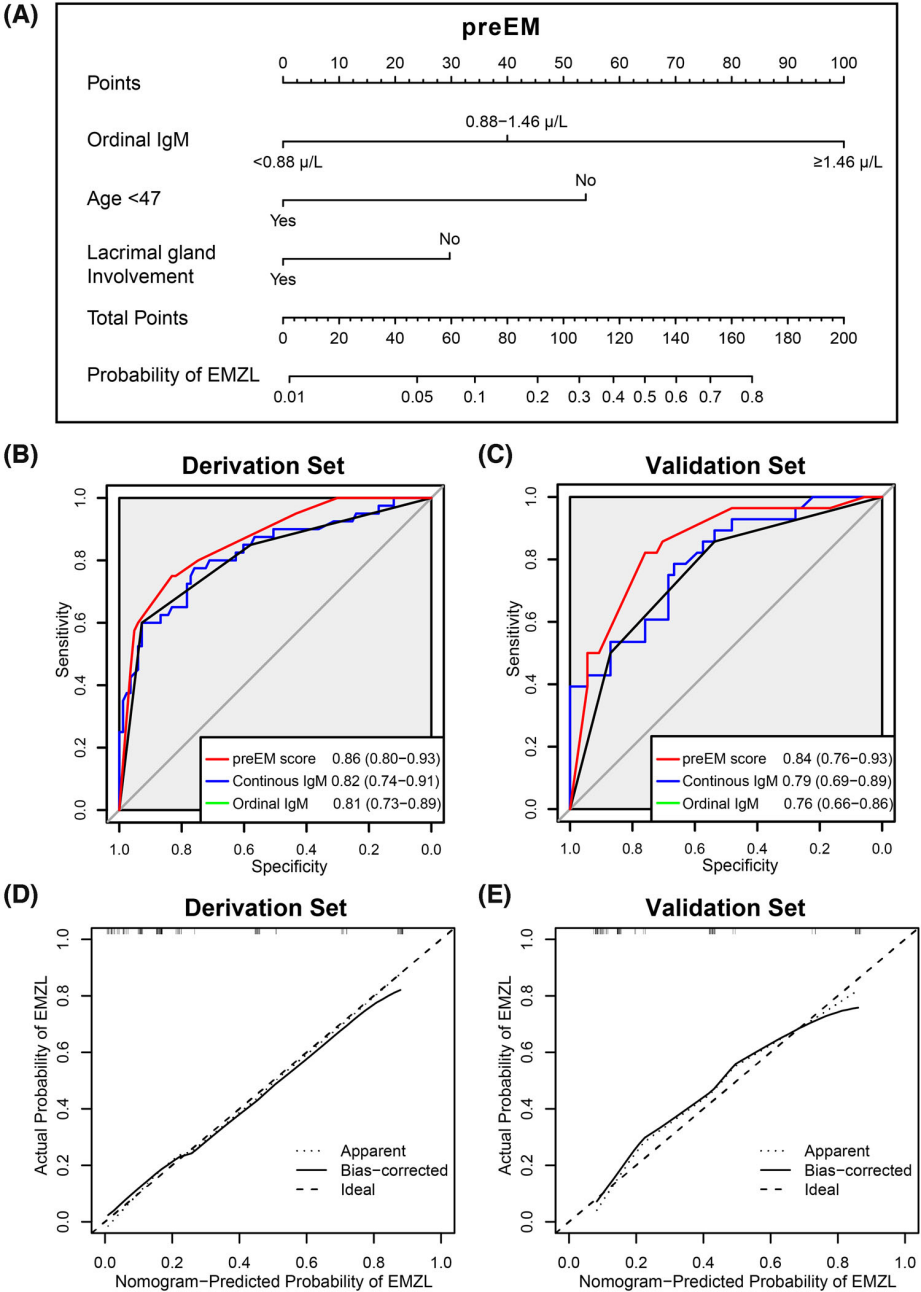
Nomogram and validation plots. (A) The nomogram entitled preliminary extranodal marginal zone lymphoma (EMZL) diagnostic model (preEM) predicting EMZL probability is developed based on three prognostic factors. Among all patients, 60% (40 EMZLs, 19 other lymphoma subtypes, 19 LHs and 45 chronic inflammations) are randomly selected as the derivation set, and the other 40% (28 EMZLs, 14 other lymphoma subtypes, seven LHs and 33 chronic inflammations) are used as the validation cohort. (B) Receiver operating characteristic (ROC) plot of the derivation set based on preEM score, continuous serum immunoglobulin M (IgM), and ordinal serum IgM. (C) ROC plot of the validation set based on preEM score, continuous serum IgM, and ordinal serum IgM. (D) Calibration plot of the derivation set. (E) Calibration plot of the validation set.

Previous findings have established the diagnostic application of diffusion‐weighted magnetic resonance imaging (MRI) for OAL.^3^ Herein, we identified preEM as a practical preoperative diagnostic model for its major subtype, OA‐EMZL. The combination of these two might provide a complete diagnostic process for orbital lymphoproliferative diseases and chronic inflammation.

Although EMZLs exhibited a high IgM, the results suggested an intertumoral heterogeneity (Figures 1G and 2A). We performed a differential expression analysis based on transcriptomic data between IgM‐high and IgM‐low EMZLs to investigate potential mechanisms (Figure S6A). The result suggested that activated GPCR, WNT and interleukin 10‐related genesets were associated with higher IgM (Figure S6B,C). Further study is required to decipher this heterogeneity and the limitation of IgM for diagnosis.

## 1 CONCLUSION

Serum IgM is a tumour‐derived biomarker used to differentiate OA‐EMZL from idiopathic orbital inflammation, IgG4 related disease and orbital lymphoproliferative disorders. The preEM constructed based on ordinal serum IgM, age at diagnosis, and lacrimal gland involvement is a practical preoperative diagnostic tool for OA‐EMZL.

## CONFLICT OF INTEREST STATEMENT

The authors declare no conflict of interest.

## FUNDING INFORMATION

This work was supported by the Project of Biobank of Shanghai Ninth People's Hospital, Shanghai Jiao Tong University School of Medicine. (grant number YBKA201907; YBKA202208). The funding organization had no role in the design or conduct of this research.

## Supporting information



Supporting Information

Supporting Information

Supporting Information
