# Supplementary material for: Multi‐omics and case‐control analyses identify immunoglobulin M as a tumour‐derived serum biomarker of ocular adnexal extranodal marginal zone lymphoma
Source: Clin Transl Med. 2023 May 3;13(5):e1259. doi: 10.1002/ctm2.1259 (PMC10157263; doi:10.1002/ctm2.1259)
Supplement: Supplementary file 1 — Supporting Information [file CTM2-13-e1259-s003.docx]

**Methods**

**Study design and ethical approval**

The study protocol was approved by the institutional review board of Ninth People's Hospital, Shanghai Jiao Tong University School of Medicine (protocol SH9H-2019-T185-2). This study was performed in accordance with the tenets of the Declaration of Helsinki, and the data were analyzed anonymously. Informed consent was obtained from patients enrolled in the proteomic and transcriptomic analysis.

**Patient selection**

We reviewed the medical records database of the Department of Ophthalmology, Ninth People's Hospital, Shanghai Jiao Tong University School of Medicine to identify patients with diagnoses of OAL, lymphoid hyperplasia (LH), and chronic orbital inflammation confirmed by surgical biopsy from January 2016 to September 2022. The pathological slides were reviewed by three pathologists to verify the diagnosis.

Inclusion criteria for patients enrolled in the proteomic and transcriptomic analysis were as follows: (1) histologically confirmed diagnosis of OAL, idiopathic orbital inflammation (IOI), reactive lymphoid hyperplasia (RLH); (2) availability of clinical and laboratory information at the time of diagnosis; and (3) specimen stored at -80℃. IOI, RLH, and normal specimens were defined as controls. The clinical characteristics of these patients are summarized in Table S1.

Inclusion criteria for the observational case series were as follows: (1) histologically confirmed diagnosis of OAL, LH, and chronic orbital inflammation (IOI, IgG4-related disease, Granuloma, Mikulicz disease, Sarcoidosis disease, Kimura disease, Sjögren syndrome, and Amyloidosis), and (2) availability of serum immunoglobin information examined by BN ProSpec System (Siemens) at the time of diagnosis. Clinical data were obtained from medical records. The clinical characteristics of these patients are summarized in Table S3.

**Protein sample preparation and sequencing**

All specimens were stored at -80 ℃ until protein isolation, and sequencing was performed by the Proteomics Platform of Core Facility of Basic Medical Sciences, Shanghai Jiao Tong University School of Medicine (SJTU-SM).

High abundant protein was removed before sequencing (A36369, ThermoScientific, USA). Samples for data-independent acquisition (DIA) mass spectrometry were denatured with 2% SDS buffer and digested with sequencing grade modified trypsin (Promega). Peptides were collected and purified using the C18 Ziptips and SpeedVac (ThermoSavant). The digested peptides were further fractionated using high pH reversed-phase separation on Dionex UHPLC (ThermoScientific, USA).

Spectral libraries were generated using data-independent acquisition (DDA) analysis with Orbitrap Fusion LUMOS mass spectrometer (Thermo Fisher Scientific) connected to Easy-nLC 1200 via an Easy Spray (Thermo Fisher Scientific). Raw data were analyzed to generate a spectral library using the Pulsar search engine (only available in Spectronaut Pulsar).

To resolve the DIA data based on the spectral library of the DDA data, the DIA MS/MS acquisition was performed with the same liquid chromatograph mass spectrometer (LC-MS) systems and linear gradient methods as DDA. The DIA raw files were analyzed in Spectronaut X (Biognosys, Switzerland) with default settings. All results were filtered by FDR cutoff of 0.01

The proteomic data have been uploaded into the iProX database (https://www.iprox.org); (project ID IPX0005210000).

Prior to analysis, low-abundance proteins and proteins missing in more than 20 percent of patients were removed. The sequential K-nearest neighbor (seqKNN) imputation method determined by NAguideR was used for missing value imputation [1].

**RNA sample preparation and sequencing**

All specimens were stored at -80 ℃ until RNA isolation, and sequencing was performed by Beijing CapitalBio Technology Inc.

RNA samples were prepared using TRIzol reagent (Ambion, 15596-026) following the manufacturer’s protocol. mRNA molecules containing Poly-A were purified from RNA using poly-T oligo-attached magnetic beads. The fragments were reversely transcribed into first strand cDNA using random hexamers, followed by synthesis of second strand cDNA using DNA polymerase I and RNase H. DNA fragments with adapter molecules on both ends were selectively enriched and amplified using PCR. The library was identified using the Agilent 2100 bioanalyzer and quantified by Qubit and qPCR. The produced libraries were sequenced on the illumina Novaseq 6000 platform. Reads were aligned to hg38. The RNA-seq data have been deposited in the Gene Expression Omnibus database (https://www.ncbi.nlm.nih.gov/geo) under accession numbers GSE216340.

Data from EMZL, diffuse large B-cell lymphoma (DLBCL), RLH, and IOI samples in our previously uploaded datasets GSE171059 and GSE199517 were combined for further analyses [2]. The combat function of sva package was used to remove the batch effect. Low-abundance transcripts were removed before analysis.

**Light chain restriction calculation**

IGLC2 was used to represent λ light chain, and IGKC to represent κ chain. We normalized light chain level before calculation. For normalization, Median IGKC/IGLC2 ratio of samples of nonmalignant diseases (IOI and RLH) was assumed as 1 considering they absent monotypic immunoglobulin and was used to multiple IGLC2 expression level. A segmental function based on different κ/λ ratio was utilized to get absolute κ/λ score and portrait the light chain restriction. The calculation process was described as follows:

$$\kappa=IGKC$$

$$\lambda_{normalized}=\left( \frac{{IGKC}_{nonmalignant}}{{IGLC2}_{nonmalignant}} \right)_{median}\times IGLC2$$

When $\kappa>\lambda_{normalized}$, $Absolute \kappa/\lambda score=\frac{\kappa}{\lambda_{normalized}} -1$

When $\lambda_{normalized}>\kappa$, $Absolute \kappa/\lambda score=\frac{\lambda_{normalized}}{\kappa} -1$

**Differential expression analysis**

Differential expression analysis was performed using limma R package (version: 3.46.0) (18) after normalization to identify differentially expressed proteins (DEPs) and differentially expressed genes (DEGs). We set |log2(foldchange)| > log2(1.5) and p-value <0.05 as the threshold for transcriptome data, and |log2(foldchange)| > log2(1.2) and p-value <0.05 as the threshold for proteome data. The Metascape tool (http://metascape.org) was used to identify biologic genesets of the selected genes.

**Immunofluorescent (IF) staining**

IF were performed following standard procedures. Five-micron thick formalin-fixed paraffin embedded (FFPE) human tissue slides were used for the experiments. Stained slides were digitized using the Pannoramic DESK (3D HISTECH) with a 40× objective lens.

Slides were stained with the MNDA antibody (#3329, CST, 1:200), IgM antibody (#66484-1-lg, Proteintech, 1:200) and CD20 antibody (#24828-1-AP, Proteintech, 1:50) following the manufacturer’s instructions.

**Definitions**

The disease stage was defined following the eighth edition of the American Joint Committee on Cancer classification and the Ann Arbor (AA) Stage [3, 4]. The international prognostic index (IPI) score was calculated as previously described [5]. ΔIgM was used to represent the change in serum IgM concentration. ΔIgM equaled post-treatment serum IgM concentration minus pre-treatment serum IgM concentration.

**Construction of preliminary EMZL diagnostic model (preEM)**

Among all patients, 60% (40 EMZLs, 19 other lymphoma subtypes, 19 LHs, and 45 chronic inflammations) were randomly selected as the derivation set, and the other 40% (28 EMZLs, 14 other lymphoma subtypes, 7 LHs, and 33 chronic inflammations) were used as the validation cohort.

The nomogram for predicting the probability of EMZL was constructed based on the results of multivariate logistic regression analysis in the derivation cohort. We calculated the results of the nomogram as: preEM score = 1.450 x (IgM concentration 0.88-1.46 g/L) + 3.625 x (IgM concentration ≥1.46 g/L) - 1.957 x (age <47) - 1.077 x (lacrimal gland involvement).

**Propensity score matching**

We performed propensity score matching (PSM) to reduce bias of cofounder factors as described [6]. The propensity score for each individual was calculated given the covariates of sex, age, date of diagnosis, bilateral involvement, and disease site. Thereafter, we applied 1:1 nearest neighbor matching to ensure minimal bias.

**Statistical analysis**

The chi-square test and the Fisher’s exact test were used to compare categorical variables; the Mann–Whitney U test was used to compare continuous variables. The median and interquartile range (IQR) were used to describe continuous variables. Receiver operating characteristic (ROC) and Univariate and multivariate logistic regression were used to assess the contribution of variables in the prediction of EMZL. Restricted cubic splines (RCS) were used to evaluate nonlinearity.

Statistical analysis was performed using IBM SPSS Statistics version 26.0 (SPSS Inc.) and R software version 4.1.1 (The R Foundation for Statistical Computing). All statistical tests were two-sided. P values <0.05 were considered statistically significant. PSM, RCS, ROC, and nomogram were conducted by using R packages “ggplot2”, “ggpubr”, “pROC”, “survminer”, “survival”, and “rms”.

**References**

1. Wang S, Li W, Hu L, et al. NAguideR: performing and prioritizing missing value imputations for consistent bottom-up proteomic analyses. Nucleic Acids Res 2020;48:e83.

2. Shi J, Zhu T, Lin H, et al. Proteotranscriptomics of ocular adnexal B-cell lymphoma reveals an oncogenic role of alternative splicing and identifies a diagnostic marker. J Exp Clin Cancer Res 2022;41:234.

3. MB A, SB E, FL G, et al. AJCC Cancer Staging Manual. 8th ed.: Springer International Publishing; 2017.

4. Carbone PP, Kaplan HS, Musshoff K, et al. Report of the Committee on Hodgkin's Disease Staging Classification. Cancer Res 1971;31:1860-1861.

5. International Non-Hodgkin's Lymphoma Prognostic Factors P. A predictive model for aggressive non-Hodgkin's lymphoma. N Engl J Med 1993;329:987-994.

6. Austin PC. Optimal caliper widths for propensity-score matching when estimating differences in means and differences in proportions in observational studies. Pharm Stat 2011;10:150-161.
