# Supplementary material for: Multi‐omics and case‐control analyses identify immunoglobulin M as a tumour‐derived serum biomarker of ocular adnexal extranodal marginal zone lymphoma
Source: Clin Transl Med. 2023 May 3;13(5):e1259. doi: 10.1002/ctm2.1259 (PMC10157263; doi:10.1002/ctm2.1259)
Supplement: Supplementary file 2 — Supporting Information [file CTM2-13-e1259-s001.docx]

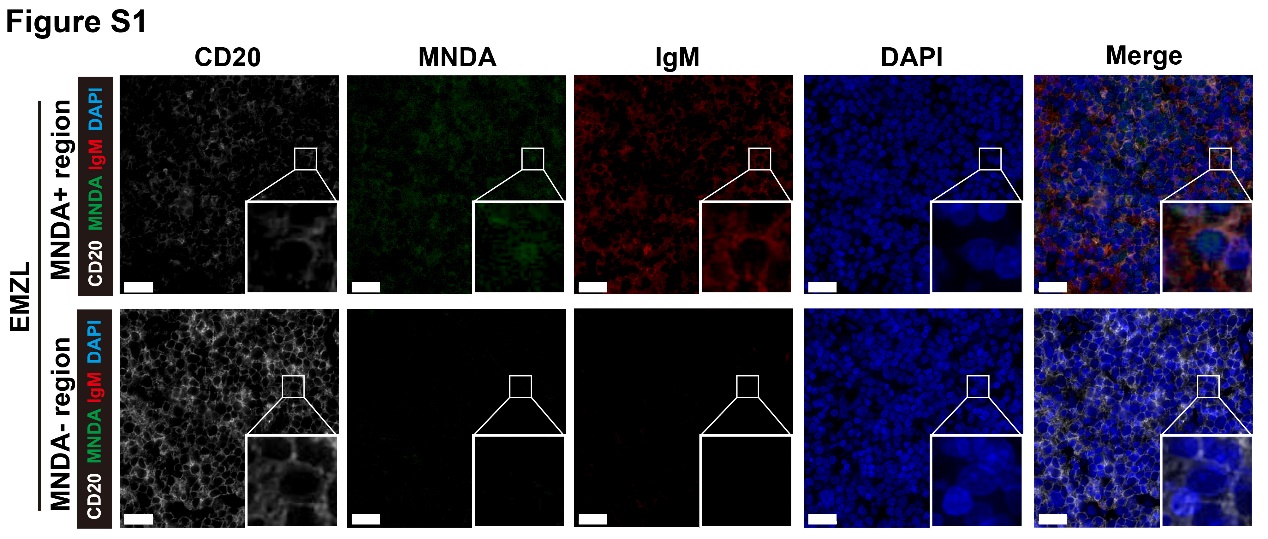
**Figure S1.** Immunofluorescent staining of OA-EMZL sample. The result shows IgM is expressed in neoplastic cells (MNDA+, CD20+) instead of bystander cells (MNDA-, CD20+).


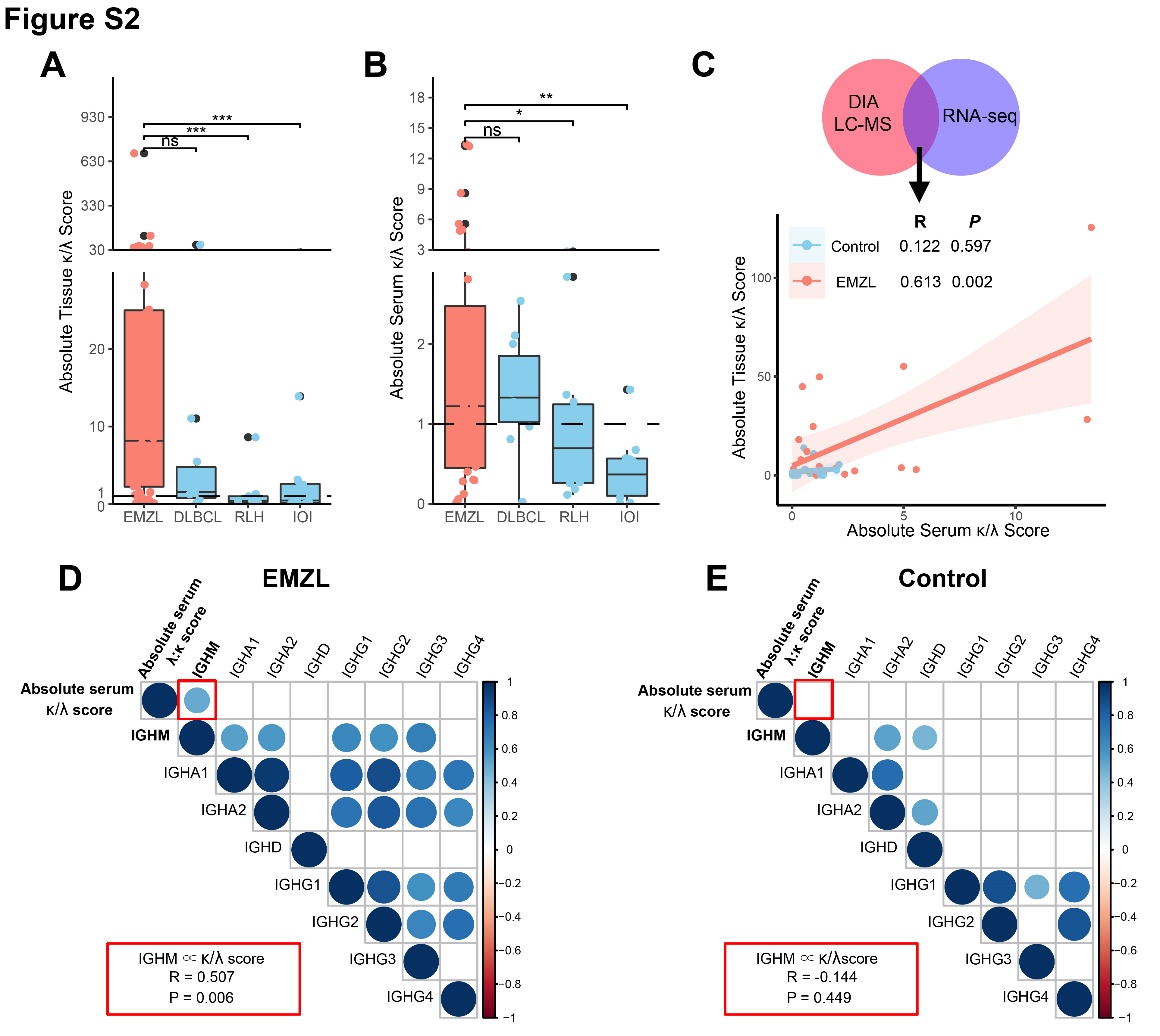


**Figure S2.** Association between IGHM and light chain restriction. (A) Boxplot shows absolute κ/λ score of subgroups calculated by tissue transcriptomic data in subgroups. Absolute tissue κ/λ score of EMZL is significantly higher than IOI and RLH. (B) Boxplot shows absolute κ/λ score of subgroups calculated by serum proteomic data. Absolute serum κ/λ score of EMZL is significantly higher than IOI and RLH. (C) Pearson correlation analysis between absolute serum κ/λ score and absolute tissue κ/λ score. We analyze tissue transcriptome and serum proteome matched samples (EMZL n =23; Control n =22). The serum score and the tissue score are significantly positively correlated in patients with EMZL and failed to be correlated in controls. (D) Pearson correlation between proteomics detected heavy chain, and absolute serum κ/λ score in EMZLs. IGHM is highly positively correlated with absolute serum κ/λ score in patients with EMZL. None of other heavy chains is significantly correlated with the score in EMZLs. (E) Pearson correlation between proteomics detected heavy chain, and absolute serum κ/λ score in controls. None of heavy chains is significantly correlated with absolute serum κ/λ score in controls.

**
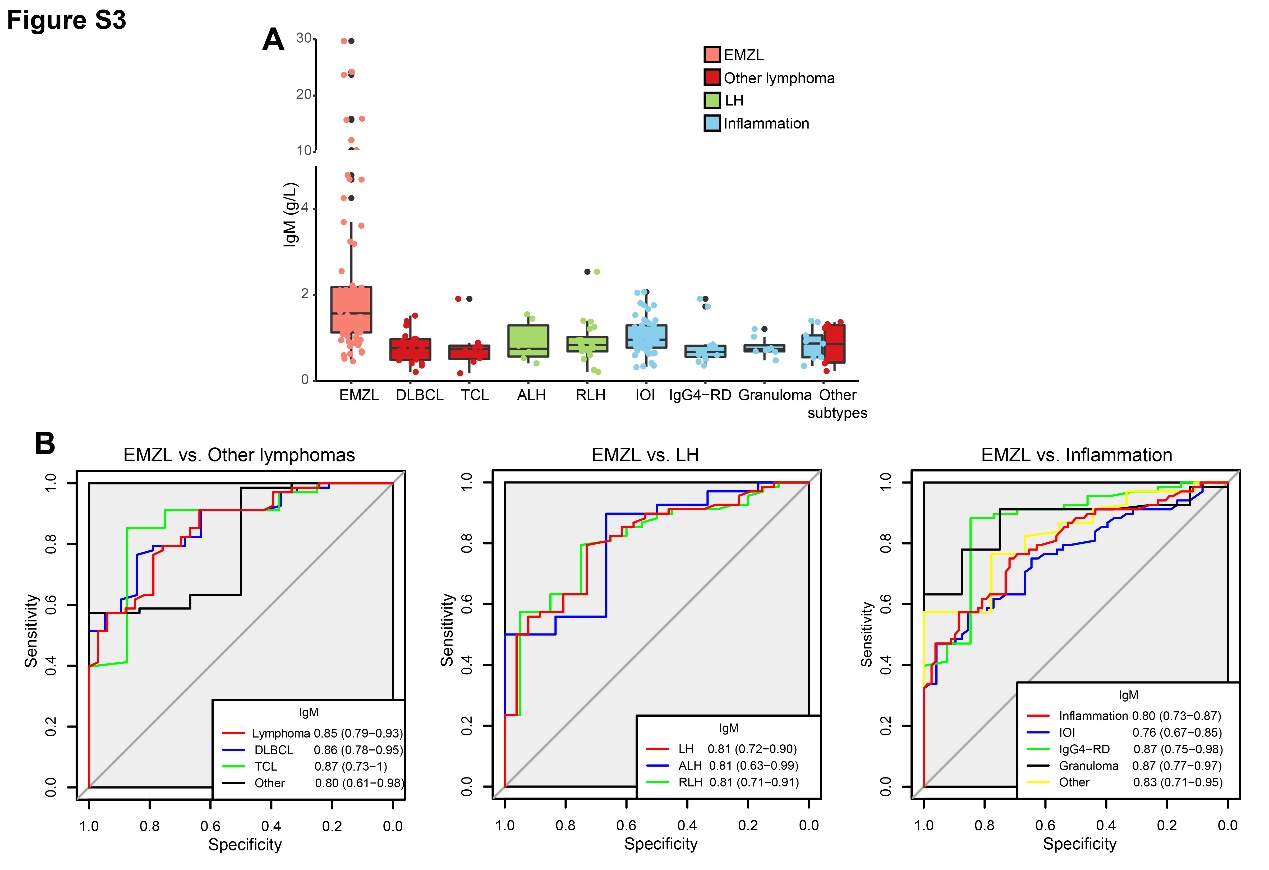
**

**Figure S3.** Serum IgM concentration across diagnoses. (A) Boxplot shows variable serum IgM concentration across diagnoses. (B) ROC plots of serum IgM in patients with EMZL and other lymphoma subtypes, patients with EMZL and LH, and patients with EMZL and chronic inflammation.


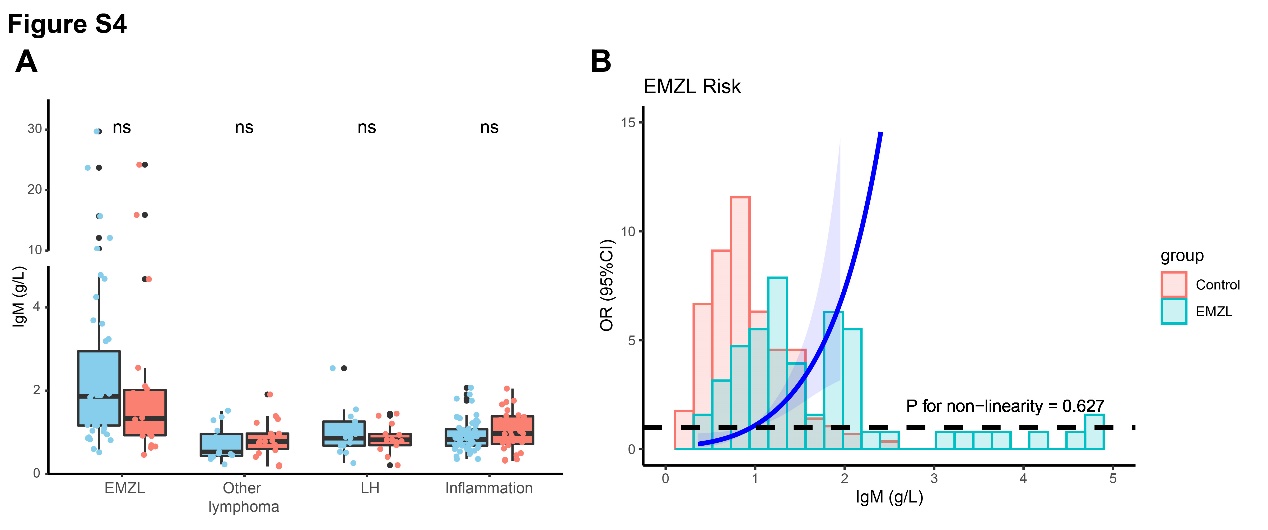


**Figure S4.** Data characteristics of serum IgM. (A) RCS analysis shows the relation of serum IgM with OR of EMZL. The vertical lines represent the knots. (B) Serum IgM is not associated with gender in EMZL, other lymphoma subtypes, LH, or chronic inflammation. “ns” indicates no statistical significance.


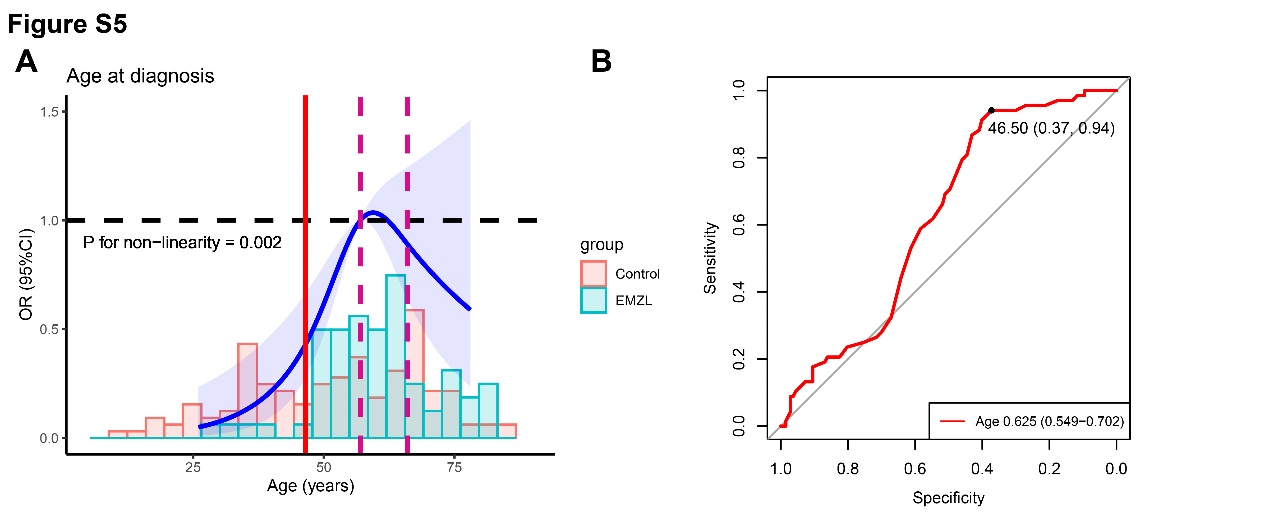


**Figure S5.** Data characteristics of age at diagnosis. (A) RCS analysis shows the relation of age with OR of EMZL. The vertical lines represent the knots. (B) ROC plot of age at diagnosis.


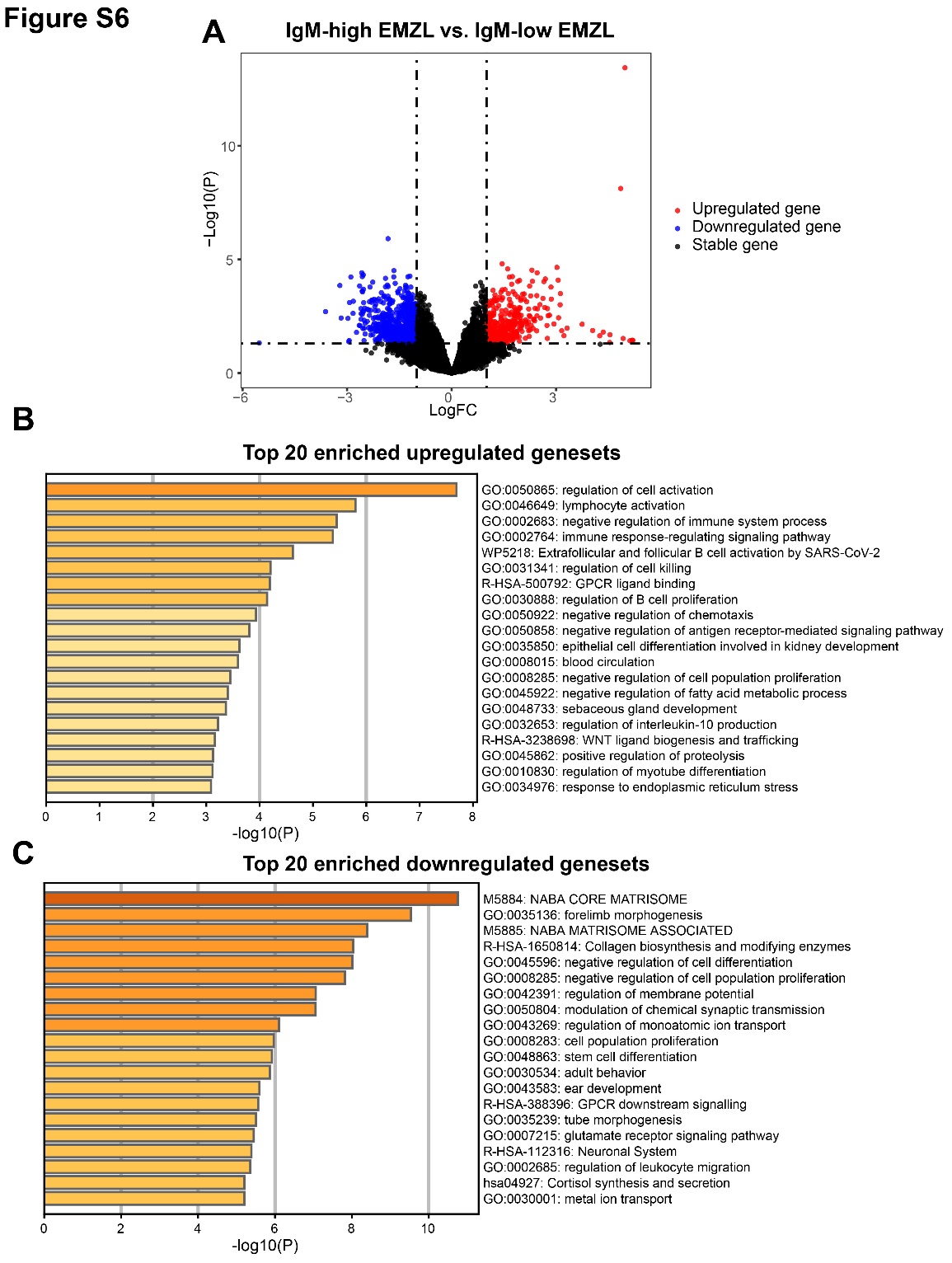


**Figure S6.** IgM reveals the intertumoral heterogeneity of EMZL. (A) Scatterplot shows differentially expressed genes (DEGs) identified in the transcriptomic cohort (38 EMZLs) between IgM-high and IgM-low samples divided by the median value of IGHM expression. The horizontal line is at P =0.05; vertical line is at transcriptomic |log2(FC)| = log2(1.5). (B) Bar plot of top 20 enrichment terms identified in upregulated genes. (C) Bar plot of top 20 enrichment terms identified in downregulated genes.
