# Supplementary material for: Multi‐omics and case‐control analyses identify immunoglobulin M as a tumour‐derived serum biomarker of ocular adnexal extranodal marginal zone lymphoma
Source: Clin Transl Med. 2023 May 3;13(5):e1259. doi: 10.1002/ctm2.1259 (PMC10157263; doi:10.1002/ctm2.1259)
Supplement: Supplementary file 3 — Supporting Information [file CTM2-13-e1259-s002.docx]

**Table S1.** Basic characteristics of patients in screening cohort. EMZL, extranodal marginal zone B-cell lymphoma; DLBCL, diffuse large B-cell lymphoma; GCB, germinal center B-cell; IOI, idiopathic orbital inflammation; RLH, reactive lymphoid hyperplasia.

| **Group** | | | **Screening** | | | | **Basic clinical feature** | | | | | | | **OAL clinical feature** | | | | | |
| --- | --- | --- | --- | --- | --- | --- | --- | --- | --- | --- | --- | --- | --- | --- | --- | --- | --- | --- | --- |
| **ID** | **Group** | **Diagnosis** | **RNAseq_GEO accession** | **Tissue RNAseq** | **Serum LC-MS** | **Matched data** | **Sex** | **Age** | **Bilateral** | **Conjunctiva** | **Eyelid** | **Lacrimal gland** | **Orbit** | **Primary OAL** | **Ann Arbor Stage** | **T** | **N** | **M** | **IPI** |
| EM_22_02 | EMZL | EMZL | GSE216340 | Yes | Yes | Yes | male | 66 | Yes | No | No | No | Yes | Yes | IV | 2 | 3 | 1b | 4 |
| EM_22_05 | EMZL | EMZL | GSE216340 | Yes | Yes | Yes | male | 74 | No | No | No | No | Yes | Yes | IIE | 2 | 1b | 0 | 1 |
| EM_22_11 | EMZL | EMZL | GSE216340 | Yes | Yes | Yes | female | 63 | No | No | No | No | Yes | Yes | IE | 2 | 0 | 0 | 1 |
| EM_22_13 | EMZL | EMZL | GSE216340 | Yes | Yes | Yes | male | 82 | No | No | No | No | Yes | Yes | IE | 2 | 0 | 0 | 1 |
| EM_22_14 | EMZL | EMZL | GSE216340 | Yes | Yes | Yes | male | 79 | No | No | No | No | Yes | Yes | IE | 2 | 0 | 0 | 1 |
| EM_22_04 | EMZL | EMZL | GSE216340 | Yes | Yes | Yes | female | 59 | No | No | No | No | Yes | Yes | IE | 2 | 0 | 0 | 0 |
| EM_22_09 | EMZL | EMZL | GSE216340 | Yes | Yes | Yes | female | 60 | No | No | No | No | Yes | Yes | IE | 2 | 0 | 0 | 1 |
| EM_22_15 | EMZL | EMZL | GSE216340 | Yes | Yes | Yes | female | 49 | No | No | No | Yes | No | Yes | IE | 2 | 0 | 0 | 0 |
| EM_22_21 | EMZL | EMZL | GSE216340 | Yes | Yes | Yes | female | 49 | No | No | Yes | Yes | No | Yes | IE | 2 | 0 | 0 | 0 |
| EM_22_16 | EMZL | EMZL | GSE216340 | Yes | Yes | Yes | female | 76 | No | No | Yes | No | Yes | Yes | IE | 3 | 0 | 0 | 1 |
| EM_22_12 | EMZL | EMZL | GSE216340 | Yes | Yes | Yes | male | 68 | Yes | Yes | No | No | Yes | No | IE | 2 | 0 | 0 | 1 |
| EM_22_19 | EMZL | EMZL | GSE216340 | Yes | Yes | Yes | male | 61 | No | No | No | Yes | Yes | No | IE | 2 | 0 | 0 | 1 |
| EM_21_27 | EMZL | EMZL | GSE171059 | Yes | Yes | Yes | male | 48 | No | No | Yes | No | No | Yes | IV | 4 | 1a | 1a | 2 |
| EM_21_03 | EMZL | EMZL | GSE171059 | Yes | Yes | Yes | female | 65 | Yes | No | No | Yes | No | Yes | IV | 2 | 0 | 1a | 3 |
| EM_21_25 | EMZL | EMZL | GSE171059 | Yes | Yes | Yes | female | 84 | No | No | No | No | Yes | Yes | IIE | 2 | 0 | 1a | 2 |
| EM_21_21 | EMZL | EMZL | GSE171059 | Yes | Yes | Yes | male | 62 | No | No | No | No | Yes | Yes | IE | 2 | 0 | 0 | 1 |
| EM_21_02 | EMZL | EMZL | GSE171059 | Yes | Yes | Yes | male | 68 | No | No | No | No | Yes | Yes | IE | 2 | 0 | 0 | 1 |
| EM_21_17 | EMZL | EMZL | GSE171059 | Yes | Yes | Yes | male | 51 | No | No | No | No | Yes | Yes | IE | 2 | 0 | 0 | 0 |
| EM_21_04 | EMZL | EMZL | GSE171059 | Yes | Yes | Yes | female | 62 | No | Yes | No | No | No | Yes | IE | 2 | 0 | 0 | 1 |
| EM_21_15 | EMZL | EMZL | GSE171059 | Yes | Yes | Yes | female | 40 | No | No | No | No | Yes | Yes | IE | 2 | 0 | 0 | 0 |
| EM_21_28 | EMZL | EMZL | GSE171059 | Yes | Yes | Yes | male | 51 | Yes | No | No | No | Yes | No | IV | 3 | 0 | 1a | 2 |
| EM_21_09 | EMZL | EMZL | GSE171059 | Yes | Yes | Yes | male | 62 | Yes | No | Yes | No | Yes | No | IE | 3 | 0 | 0 | 1 |
| EM_21_26 | EMZL | EMZL | GSE171059 | Yes | Yes | Yes | female | 72 | No | No | No | No | Yes | Yes | IE | 2 | 0 | 0 | 1 |
| EM_22_03 | EMZL | EMZL | GSE216340 | Yes | No | No | male | 71 | No | No | No | No | Yes | Yes | IE | 2 | 0 | 0 | 1 |
| EM_22_20 | EMZL | EMZL | GSE216340 | Yes | No | No | male | 36 | No | No | No | No | Yes | Yes | IE | 2 | 0 | 0 | 0 |
| EM_22_07 | EMZL | EMZL | GSE216340 | Yes | No | No | female | 52 | No | No | No | No | Yes | Yes | IE | 2 | 0 | 0 | 0 |
| EM_22_17 | EMZL | EMZL | GSE216340 | Yes | No | No | female | 53 | No | No | No | No | Yes | Yes | IE | 2 | 0 | 0 | 0 |
| EM_22_18 | EMZL | EMZL | GSE216340 | Yes | No | No | male | 51 | No | No | No | No | Yes | Yes | IE | 2 | 0 | 0 | 0 |
| EM_21_14 | EMZL | EMZL | GSE171059 | Yes | No | No | male | 45 | No | No | No | Yes | Yes | Yes | IIIE | 2 | 0 | 1a | 2 |
| EM_21_22 | EMZL | EMZL | GSE171059 | Yes | No | No | male | 59 | No | No | No | No | Yes | Yes | IE | 2 | 0 | 0 | 0 |
| EM_21_20 | EMZL | EMZL | GSE171059 | Yes | No | No | male | 73 | No | No | No | No | Yes | Yes | IE | 2 | 0 | 0 | 1 |
| EM_21_16 | EMZL | EMZL | GSE171059 | Yes | No | No | male | 81 | No | No | No | No | Yes | Yes | IE | 2 | 0 | 0 | 1 |
| EM_21_06 | EMZL | EMZL | GSE171059 | Yes | No | No | female | 67 | No | No | No | No | Yes | Yes | IE | 2 | 0 | 0 | 1 |
| EM_21_18 | EMZL | EMZL | GSE171059 | Yes | No | No | female | 72 | Yes | No | Yes | No | Yes | Yes | IE | 3 | 0 | 0 | 1 |
| EM_21_13 | EMZL | EMZL | GSE171059 | Yes | No | No | female | 82 | No | No | No | No | Yes | Yes | IE | 2 | 0 | 0 | 1 |
| EM_21_11 | EMZL | EMZL | GSE171059 | Yes | No | No | female | 65 | No | No | No | No | Yes | Yes | IE | 2 | 0 | 0 | 1 |
| EM_21_23 | EMZL | EMZL | GSE171059 | Yes | No | No | male | 64 | No | No | No | No | Yes | Yes | IE | 2 | 0 | 0 | 1 |
| EM_21_08 | EMZL | EMZL | GSE171059 | Yes | No | No | male | 46 | No | No | No | No | Yes | No | IE | 2 | 0 | 0 | 0 |
| EM_21_24 | EMZL | EMZL | GSE171059 | Yes | No | No | male | 64 | No | No | No | No | Yes | No | IE | 2 | 0 | 0 | 1 |
| EM_22_01 | EMZL | EMZL |  | No | Yes | No | male | 63 | Yes | No | No | No | Yes | Yes | IE | 2 | 0 | 0 | 1 |
| EM_22_24 | EMZL | EMZL |  | No | Yes | No | female | 57 | No | No | No | No | Yes | Yes | IE | 2 | 0 | 0 | 0 |
| EM_22_25 | EMZL | EMZL |  | No | Yes | No | male | 57 | Yes | No | No | No | Yes | Yes | IE | 2 | 1b | 0 | 0 |
| EM_22_06 | EMZL | EMZL |  | No | Yes | No | male | 63 | No | No | No | Yes | Yes | Yes | IE | 2 | 0 | 0 | 1 |
| EM_22_26 | EMZL | EMZL |  | No | Yes | No | female | 60 | No | Yes | No | No | No | No | IV | 1 | 1 | 1a | 2 |
| DB_22_01 | Control | DLBCL, CD5+ |  | No | Yes | No | female | 56 | No | No | No | No | Yes | Yes | IV | 4 | 1b | 0 | 2 |
| DB_22_02 | Control | DLBCL, GCB | GSE216340 | Yes | Yes | Yes | male | 68 | No | No | No | No | Yes | Yes | IE | 2 | 0 | 0 | 3 |
| DB_21_04 | Control | DLBCL, GCB | GSE171059 | Yes | Yes | Yes | male | 81 | No | No | No | No | Yes | Yes | IV | 4 | 0 | 0 | 4 |
| DB_22_04 | Control | DLBCL, nonGCB | GSE216340 | Yes | Yes | Yes | female | 58 | No | No | No | Yes | Yes | Yes | IIE | 4 | 1b | 0 | 1 |
| DB_22_03 | Control | DLBCL, nonGCB | GSE216340 | Yes | Yes | Yes | male | 77 | No | No | No | No | Yes | Yes | IE | 0 | 0 | 0 | 2 |
| DB_21_01 | Control | DLBCL, nonGCB | GSE171059 | Yes | Yes | Yes | male | 75 | No | No | No | No | Yes | Yes | IIE | 4 | 0 | 0 | 2 |
| DB_21_02 | Control | DLBCL, nonGCB | GSE171059 | Yes | Yes | Yes | female | 66 | No | No | No | No | Yes | Yes | IIE | 4 | 1b | 0 | 3 |
| DB_21_07 | Control | DLBCL, nonGCB | GSE171059 | Yes | Yes | Yes | male | 68 | No | No | No | Yes | No | Yes | IE | 2 | 0 | 0 | 1 |
| DB_21_08 | Control | DLBCL, nonGCB | GSE171059 | Yes | Yes | Yes | female | 69 | Yes | No | No | No | Yes | Yes | IE | 2 | 0 | 0 | 2 |
| DB_21_03 | Control | DLBCL, nonGCB | GSE171059 | Yes | No | No | male | 64 | No | No | No | No | Yes | Yes | IV | 4 | 0 | 0 | 4 |
| DB_21_05 | Control | DLBCL, nonGCB | GSE171059 | Yes | No | No | female | 51 | Yes | No | No | Yes | Yes | Yes | IV | 2 | 0 | 1a | 3 |
| DB_22_05 | Control | DLBCL, nonGCB |  | No | Yes | No | male | 76 | No | No | No | No | Yes | Yes | IE | 2 | 0 | 0 | 4 |
| OI_22_04 | Control | IOI | GSE216340 | Yes | Yes | Yes | male | 67 | No | No | No | No | Yes |  |  |  |  |  |  |
| OI_22_02 | Control | IOI | GSE216340 | Yes | Yes | Yes | male | 60 | No | No | No | No | Yes |  |  |  |  |  |  |
| OI_22_03 | Control | IOI | GSE216340 | Yes | Yes | Yes | male | 50 | No | No | No | No | Yes |  |  |  |  |  |  |
| OI_22_05 | Control | IOI | GSE216340 | Yes | Yes | Yes | female | 44 | No | No | No | Yes | Yes |  |  |  |  |  |  |
| OI_21_09 | Control | IOI | GSE199517 | Yes | Yes | Yes | female | 60 | Yes | No | No | Yes | No |  |  |  |  |  |  |
| OI_21_03 | Control | IOI | GSE199517 | Yes | Yes | Yes | female | 74 | No | No | No | No | Yes |  |  |  |  |  |  |
| OI_21_01 | Control | IOI | GSE199517 | Yes | Yes | Yes | female | 34 | No | No | Yes | No | No |  |  |  |  |  |  |
| OI_21_08 | Control | IOI | GSE199517 | Yes | No | No | male | 56 | Yes | No | No | No | Yes |  |  |  |  |  |  |
| OI_21_07 | Control | IOI | GSE199517 | Yes | No | No | female | 43 | No | No | No | Yes | Yes |  |  |  |  |  |  |
| OI_21_04 | Control | IOI | GSE199517 | Yes | No | No | female | 60 | No | No | Yes | No | No |  |  |  |  |  |  |
| OI_21_02 | Control | IOI | GSE199517 | Yes | No | No | female | 48 | No | No | No | No | Yes |  |  |  |  |  |  |
| OI_21_13 | Control | IOI | GSE199517 | Yes | No | No | female | 50 | No | No | No | Yes | Yes |  |  |  |  |  |  |
| OI_21_11 | Control | IOI | GSE199517 | Yes | No | No | male | 23 | No | No | No | No | Yes |  |  |  |  |  |  |
| OI_22_07 | Control | IOI |  | No | Yes | No | male | 86 | No | No | No | No | Yes |  |  |  |  |  |  |
| OI_22_10 | Control | IOI |  | No | Yes | No | female | 50 | No | No | Yes | No | No |  |  |  |  |  |  |
| OI_22_01 | Control | IOI |  | No | Yes | No | female | 35 | No | No | No | No | Yes |  |  |  |  |  |  |
| LP_22_06 | Control | RLH | GSE216340 | Yes | Yes | Yes | male | 56 | No | No | No | No | Yes |  |  |  |  |  |  |
| LP_22_08 | Control | RLH | GSE216340 | Yes | Yes | Yes | female | 68 | No | No | No | Yes | No |  |  |  |  |  |  |
| LP_22_09 | Control | RLH | GSE216340 | Yes | Yes | Yes | female | 57 | Yes | No | No | Yes | No |  |  |  |  |  |  |
| LP_22_05 | Control | RLH | GSE216340 | Yes | Yes | Yes | female | 53 | No | Yes | No | No | No |  |  |  |  |  |  |
| LP_22_04 | Control | RLH | GSE216340 | Yes | Yes | Yes | female | 45 | Yes | No | No | No | Yes |  |  |  |  |  |  |
| LP_22_07 | Control | RLH | GSE216340 | Yes | Yes | Yes | male | 54 | No | No | No | No | Yes |  |  |  |  |  |  |
| LP_21_02 | Control | RLH | GSE199517 | Yes | Yes | Yes | female | 80 | No | No | Yes | No | No |  |  |  |  |  |  |
| LP_21_03 | Control | RLH | GSE199517 | Yes | No | No | female | 74 | Yes | No | No | No | Yes |  |  |  |  |  |  |
| LP_21_01 | Control | RLH | GSE199517 | Yes | No | No | male | 69 | No | No | No | No | Yes |  |  |  |  |  |  |
| LP_22_01 | Control | RLH |  | No | Yes | No | male | 39 | Yes | No | No | Yes | Yes |  |  |  |  |  |  |
| LP_22_02 | Control | RLH |  | No | Yes | No | male | 76 | No | No | No | No | Yes |  |  |  |  |  |  |
| LP_22_03 | Control | RLH |  | No | Yes | No | male | 66 | Yes | No | No | No | Yes |  |  |  |  |  |  |

**Table S2.** Immunoglobulin light chain staining of patients in screening cohort. EMZL, extranodal marginal zone B-cell lymphoma; DLBCL, diffuse large B-cell lymphoma; GCB, germinal center B-cell; IOI, idiopathic orbital inflammation; RLH, reactive lymphoid hyperplasia.

| **Group** | | | **IHC immunoglobulin light chain staining** | |
| --- | --- | --- | --- | --- |
| **ID** | **Group** | **Diagnosis** | **Kappa** | **Lambda** |
| EM_21_02 | EMZL | EMZL | partial + | partial + |
| EM_21_03 | EMZL | EMZL | + | partial + |
| EM_21_04 | EMZL | EMZL | - | + |
| EM_21_06 | EMZL | EMZL | - | + |
| EM_21_08 | EMZL | EMZL | partial + | low + |
| EM_21_09 | EMZL | EMZL | partial + | low + |
| EM_21_11 | EMZL | EMZL | + | low + |
| EM_21_13 | EMZL | EMZL | + | partial + |
| EM_21_14 | EMZL | EMZL | low + | + |
| EM_21_15 | EMZL | EMZL | partial + | + |
| EM_21_16 | EMZL | EMZL | + | low + |
| EM_21_17 | EMZL | EMZL | - | - |
| EM_21_18 | EMZL | EMZL | low + | + |
| EM_21_20 | EMZL | EMZL | partial + | + |
| EM_21_21 | EMZL | EMZL | - | + |
| EM_21_22 | EMZL | EMZL | - | + |
| EM_21_23 | EMZL | EMZL | partial + | partial + |
| EM_21_24 | EMZL | EMZL | + | partial + |
| EM_21_25 | EMZL | EMZL | partial + | + |
| EM_21_26 | EMZL | EMZL | partial + | partial + |
| EM_21_27 | EMZL | EMZL | + | partial + |
| EM_21_28 | EMZL | EMZL | - | + |
| EM_22_01 | EMZL | EMZL | - | + |
| EM_22_02 | EMZL | EMZL | partial + | + |
| EM_22_03 | EMZL | EMZL | partial + | + |
| EM_22_04 | EMZL | EMZL | partial + | + |
| EM_22_05 | EMZL | EMZL | - | + |
| EM_22_06 | EMZL | EMZL | - | + |
| EM_22_07 | EMZL | EMZL | + | partial + |
| EM_22_09 | EMZL | EMZL | - | + |
| EM_22_11 | EMZL | EMZL | partial + | partial + |
| EM_22_12 | EMZL | EMZL | - | + |
| EM_22_13 | EMZL | EMZL | partial + | partial + |
| EM_22_14 | EMZL | EMZL | - | + |
| EM_22_15 | EMZL | EMZL | - | partial + |
| EM_22_16 | EMZL | EMZL | partial + | + |
| EM_22_17 | EMZL | EMZL | partial + | partial + |
| EM_22_18 | EMZL | EMZL | + | - |
| EM_22_19 | EMZL | EMZL | partial + | - |
| EM_22_20 | EMZL | EMZL | + | - |
| EM_22_21 | EMZL | EMZL | + | partial + |
| EM_22_24 | EMZL | EMZL | + | partial + |
| EM_22_25 | EMZL | EMZL | partial + | partial + |
| EM_22_26 | EMZL | EMZL | - | partial + |
| DB_22_01 | Control | DLBCL,CD5+ | partial + | + |
| DB_21_04 | Control | DLBCL,GCB | partial + | + |
| DB_22_02 | Control | DLBCL,GCB | + | partial + |
| DB_21_01 | Control | DLBCL,NonGCB | - | + |
| DB_21_02 | Control | DLBCL,NonGCB | + | partial + |
| DB_21_03 | Control | DLBCL,NonGCB | partial + | partial + |
| DB_21_05 | Control | DLBCL,NonGCB | partial + | + |
| DB_21_07 | Control | DLBCL,NonGCB | - | + |
| DB_21_08 | Control | DLBCL,NonGCB | + | partial + |
| DB_22_03 | Control | DLBCL,NonGCB | partial + | + |
| DB_22_04 | Control | DLBCL,NonGCB | partial + | + |
| DB_22_05 | Control | DLBCL,NonGCB | - | + |
| OI_21_01 | Control | IOI | partial + | partial + |
| OI_21_02 | Control | IOI | partial + | partial + |
| OI_21_03 | Control | IOI | low + | low + |
| OI_21_04 | Control | IOI | low + | low + |
| OI_21_07 | Control | IOI | partial + | partial + |
| OI_21_08 | Control | IOI | partial + | partial + |
| OI_21_09 | Control | IOI | partial + | partial + |
| OI_21_11 | Control | IOI | low + | low + |
| OI_21_13 | Control | IOI | partial + | partial + |
| OI_22_01 | Control | IOI | partial + | partial + |
| OI_22_02 | Control | IOI | low + | low + |
| OI_22_03 | Control | IOI | partial + | partial + |
| OI_22_04 | Control | IOI | low + | low + |
| OI_22_05 | Control | IOI | partial + | partial + |
| OI_22_07 | Control | IOI | partial + | partial + |
| OI_22_10 | Control | IOI | low + | low + |
| LP_21_01 | Control | RLH | partial + | partial + |
| LP_21_02 | Control | RLH | partial + | partial + |
| LP_21_03 | Control | RLH | partial + | partial + |
| LP_22_01 | Control | RLH | + | low + |
| LP_22_02 | Control | RLH | low + | low + |
| LP_22_03 | Control | RLH | low + | partial + |
| LP_22_04 | Control | RLH | partial + | partial + |
| LP_22_05 | Control | RLH | low + | low + |
| LP_22_06 | Control | RLH | partial + | partial + |
| LP_22_07 | Control | RLH | partial + | + |
| LP_22_08 | Control | RLH | low + | low + |
| LP_22_09 | Control | RLH | partial + | partial + |

**Table S3.** Differentially expressed protein-mRNA pairs in both omics between EMZLs and controls.

|  | **RNA_log2FC** | **RNA_P** | **PRO_log2FC** | **PRO_P** | **Dysregulation** |
| --- | --- | --- | --- | --- | --- |
| IGHM | 3.15311265 | 5.635E-08 | 2.635774 | 1.38E-09 | concordant |
| SIGLEC5 | 2.00738117 | 3.176E-06 | -0.40136 | 0.012796 | disconcordant |
| SEMA7A | 1.96708623 | 1.926E-09 | -0.51104 | 0.014246 | disconcordant |
| RALGPS2 | 1.95787345 | 1.572E-12 | -0.26447 | 0.044699 | disconcordant |
| SERPINA5 | 1.916792 | 1.54E-05 | 0.292994 | 0.02045 | concordant |
| PEBP4 | 1.8552 | 0.000186 | 0.5288 | 0.001805 | concordant |
| LTF | 1.780794 | 0.0094686 | 0.582466 | 0.010774 | concordant |
| KRT2 | 1.436221 | 0.0009954 | 0.947665 | 0.001778 | concordant |
| ICAM3 | 1.375521 | 8.353E-05 | 0.334299 | 0.031522 | concordant |
| ITIH1 | 0.966183 | 0.0029474 | -0.37939 | 0.000173 | disconcordant |
| HMCN2 | 0.9473442 | 0.0058933 | 0.282331 | 0.017983 | concordant |
| IGFBP3 | 0.7360358 | 0.0041082 | 0.41598 | 0.000257 | concordant |
| ROBO1 | 0.7266565 | 0.0005059 | 0.289159 | 0.009725 | concordant |
| AHCY | -0.5897578 | 8.806E-05 | 0.372598 | 0.036432 | disconcordant |
| SPARCL1 | -0.6349554 | 0.009968 | -0.3212 | 0.026799 | concordant |
| QSOX1 | -0.6521705 | 2.094E-06 | 0.387489 | 0.000112 | disconcordant |
| LGALS3BP | -0.7556219 | 0.0001592 | 0.345279 | 0.042349 | disconcordant |
| CHGA | -0.7660175 | 0.0373599 | -1.0968 | 0.029185 | concordant |
| CDH5 | -0.7689078 | 0.0192961 | 0.420828 | 0.003346 | disconcordant |
| QPCT | -0.7840512 | 0.0011296 | 1.166617 | 0.004804 | disconcordant |
| DOCK4 | -0.8406109 | 0.0002721 | -0.34101 | 0.028524 | concordant |
| AGRN | -0.8524781 | 0.0004136 | -0.70492 | 0.024408 | concordant |
| SELENBP1 | -0.8525816 | 0.0106565 | 0.555885 | 0.01596 | disconcordant |
| CTSD | -0.8606884 | 5.492E-06 | 0.344588 | 0.012724 | disconcordant |
| SASH1 | -0.8664128 | 0.0009538 | -0.56984 | 0.005385 | concordant |
| TIE1 | -0.9030069 | 0.0007913 | 0.303596 | 0.019553 | disconcordant |
| C4BPB | -0.9072923 | 0.0335294 | -0.57228 | 0.000558 | concordant |
| LGALS3 | -0.9298304 | 1.593E-07 | 0.308488 | 0.01195 | disconcordant |
| NRP1 | -0.9302623 | 0.0002876 | 0.393717 | 0.009245 | disconcordant |
| KRT81 | -0.9474467 | 0.0430937 | -0.85606 | 0.00485 | concordant |
| COL6A2 | -0.9504024 | 2.341E-05 | 0.975709 | 0.007957 | disconcordant |
| RNASE2 | -0.9638703 | 0.0176594 | -0.55269 | 0.005534 | concordant |
| OAF | -0.9950915 | 7.05E-05 | 0.405576 | 0.00779 | disconcordant |
| JUP | -1.000279 | 0.0009615 | -0.3767 | 0.042267 | concordant |
| ITGA2 | -1.023677 | 0.0048236 | 0.293078 | 0.017894 | disconcordant |
| GOLM1 | -1.0474148 | 3.917E-07 | -0.30926 | 0.025277 | concordant |
| BCAM | -1.058789 | 0.0071989 | 0.592606 | 0.005144 | disconcordant |
| A2M | -1.087126 | 7.079E-06 | 0.484373 | 0.025769 | disconcordant |
| CRISP3 | -1.117283 | 0.0050415 | -0.94587 | 0.001309 | concordant |
| CRTAC1 | -1.144049 | 0.0073993 | 0.351027 | 0.004447 | disconcordant |
| APOD | -1.167411 | 0.0115191 | -0.6305 | 0.027453 | concordant |
| IGHA2 | -1.197754 | 0.0339941 | 0.912309 | 0.001032 | disconcordant |
| KRT6B | -1.19878 | 0.0272983 | 0.729495 | 0.020635 | disconcordant |
| IGLC2 | -1.220381 | 0.0399662 | 1.215829 | 0.000374 | disconcordant |
| SERPINA1 | -1.336782 | 1.225E-05 | 0.488869 | 0.015796 | disconcordant |
| CD248 | -1.358534 | 4.017E-05 | -0.28562 | 0.049956 | concordant |
| HBD | -1.442729 | 0.0007613 | 1.183035 | 0.019585 | disconcordant |
| IGHV3-49 | -1.495057 | 0.0321752 | 0.543518 | 0.015837 | disconcordant |
| RNASE1 | -1.539272 | 6.472E-06 | -0.42678 | 0.00347 | concordant |
| CD5L | -1.564513 | 0.0125799 | 0.713327 | 0.000605 | disconcordant |
| FAM20C | -1.7551542 | 3.035E-09 | 0.544068 | 0.032668 | disconcordant |
| LRG1 | -1.801141 | 1.518E-05 | 0.703164 | 0.000816 | disconcordant |
| IGLC3 | -1.903559 | 0.0076616 | 1.215829 | 0.000374 | disconcordant |
| IGLV3-21 | -2.006563 | 0.0110635 | 0.570844 | 0.007094 | disconcordant |
| LYVE1 | -2.166427 | 0.0004468 | 0.432294 | 0.016191 | disconcordant |
| S100A9 | -3.2737942 | 5.637E-11 | -0.56874 | 0.014042 | concordant |
| S100A8 | -3.8132377 | 7.747E-09 | -0.50853 | 0.045428 | concordant |

**Table S4.** Clinical features of patients. ‡, compared by Fisher’s exact test. no, number; IQR, interquartile range; EMZL, extranodal marginal zone B-cell lymphoma; DLBCL, diffuse large B-cell lymphoma; TCL, T-cell lymphoma; SLL, Small lymphocytic lymphoma; FL, follicular lymphoma; MCL, mantle cell lymphoma; RLH, reactive lymphoid hyperplasia; ALH, Atypical lymphoid hyperplasia; IOI, idiopathic orbital inflammation; IgG4-RD, IgG4-related disease.

|  | **Control** | **EMZL** | ***P*** |
| --- | --- | --- | --- |
| **No. of patients** | 137 | 68 |  |
| **Sex** |  |  | 0.168 |
| Male, no. (%) | 79 (57.7) | 46 (68) |  |
| Female, no. (%) | 58 (42.3) | 22 (32) |  |
| **Age at diagnosis, median. (IQR)** | 55 (38-67) | 60 (53-66.75) | **0.007** |
| **Date of diagnosis** |  |  | 0.472 |
| -2018, no. (%) | 28 (20.4) | 19 (28) |  |
| 2019-2020, no. (%) | 60 (43.8) | 26 (38) |  |
| 2021-2022, no. (%) | 49 (35.8) | 23 (34) |  |
| **Diagnosis** |  |  |  |
| **Lymphoma, no. (%)** | 33 (24.1) | 68 (100) |  |
| EMZL, no. (%) | - | 68 (100) |  |
| DLBCL, no. (%) | 19 (13.9) | - |  |
| TCL, no. (%) | 8 (5.8) | - |  |
| CLL/SLL, no. (%) | 3 (2.2) | - |  |
| FL, no. (%) | 2 (1.5) | - |  |
| MCL, no. (%) | 1 (0.7) | - |  |
| **Lymphoid hyperplasia, no. (%)** | 26 (19) | - |  |
| RLH, no. (%) | 20 (14.6) | - |  |
| ALH, no. (%) | 6 (4.4) | - |  |
| **Chronic inflammation, no. (%)** | 78 (56.9) | - |  |
| IOI, no. (%) | 48 (35) | - |  |
| IgG4-RD, no. (%) | 13 (9.5) | - |  |
| Granuloma, no. (%) | 8 (5.8) | - |  |
| Mikulicz disease, no. (%) | 4 (2.9) | - |  |
| Sarcoidosis disease, no. (%) | 2 (1.5) | - |  |
| Kimura disease, no. (%) | 1 (0.7) | - |  |
| Sjögren syndrome, no. (%) | 1 (0.7) | - |  |
| Amyloidosis, no. (%) | 1 (0.7) | - |  |
| **Anatomic site** |  |  |  |
| Orbit, no. (%) | 85 (62) | 54 (79) | **0.012** |
| Lacrimal gland, no. (%) | 45 (32.8) | 11 (16) | **0.012** |
| Eyelid, no. (%) | 27 (19.7) | 12 (18) | 0.723 |
| Conjunctiva, no. (%) | 5 (3.6) | 6 (9) | 0.185 ‡ |
| **Bilateral involvement, no. (%)** | 43 (31.4) | 15 (22) | 0.163 |

**Table S5.** Univariate and multivariate logistic analyses of EMZL. EMZL, extranodal marginal zone B-cell lymphoma; OR, odd ratio; CI, confidence interval.

|  | **EMZL** | | | |
| --- | --- | --- | --- | --- |
|  | **Univariate** | | **Multivariate** | |
| **Variables** | **OR (95%CI)** | ***P*** | **OR (95%CI)** | ***P*** |
| Serum IgM concentration | 7.48 (3.70 - 15.12) | **<0.001** | 8.609 (4.056 - 18.274) | **<0.001** |
| Age at diagnosis | 1.04 (1.02 - 1.06) | **0.001** | 1.044 (1.017 - 1.071) | **0.001** |
| Orbital involvement | 2.36 (1.19 - 4.67) | **0.014** |  |  |
| Lacrimal gland involvement | 0.395 (0.189 - 0.825) | 0.013 |  |  |
| Bilateral involvement | 0.619 (0.314 1.218) | 0.165 |  |  |
| Female sex | 0.651 (0.354 - 1.2) | 0.169 |  |  |

**Table S6.** Clinical features of patients after propensity score matching. no, number; IQR, interquartile range; EMZL, extranodal marginal zone B-cell lymphoma; DLBCL, diffuse large B-cell lymphoma; TCL, T-cell lymphoma; SLL, Small lymphocytic lymphoma; FL, follicular lymphoma; MCL, mantle cell lymphoma; RLH, reactive lymphoid hyperplasia; ALH, Atypical lymphoid hyperplasia; IOI, idiopathic orbital inflammation; IgG4-RD, IgG4-related disease.

|  | **Control** | **Case** | ***P*** |
| --- | --- | --- | --- |
| **No. of patients** | 68 | 68 |  |
| **Sex** |  |  | 0.717 |
| Male, no. (%) | 44 (64.7) | 46 (68) |  |
| Female, no. (%) | 24 (35.3) | 22 (32) |  |
| **Age at diagnosis, median. (IQR)** | 64 (52.3-70.8) | 60 (53.0-66.8) |  |
| **Date of diagnosis** |  |  | 0.453 |
| -2018, no. (%) | 31 (45.6) | 26 (38) |  |
| 2019-2020, no. (%) | 24 (35.3) | 23 (34) |  |
| 2021-2022, no. (%) | 13 (19.1) | 19 (28) |  |
| **Diagnosis** |  |  |  |
| **Lymphoma** | 25 (36.8) | 68 (100) |  |
| EMZL | - | 68 (100) |  |
| DLBCL | 15 (22.1) | - |  |
| TCL | 4 (5.9) | - |  |
| SLL | 3 (4.4) | - |  |
| FL | 2 (2.9) | - |  |
| MCL | 1 (1.5) | - |  |
| **Lymphoid hyperplasia** | 15 (22.1) | - |  |
| RLH | 12 (17.6) | - |  |
| ALH | 3 (4.4) | - |  |
| **Chronic inflammation** | 28 (41.2) | - |  |
| IOI | 18 (26.5) | - |  |
| IgG4-RD | 4 (5.9) | - |  |
| Granuloma | 4 (5.9) | - |  |
| Sarcoidosis disease | 1 (1.5) | - |  |
| Amyloidosis | 1 (1.5) | - |  |
| **Anatomic site** |  |  |  |
| Orbit, no. (%) | 54 (79.4) | 54 (79) | 1 |
| Lacrimal gland, no. (%) | 12 (17.6) | 11 (16) | 0.819 |
| Eyelid, no. (%) | 11 (16.2) | 12 (18) | 0.819 |
| Conjunctiva, no. (%) | 5 (7.4) | 6 (9) | 0.753 |
| **Bilateral involvement** | 13 (19.1) | 15 (22) | 0.671 |

**Table S7**. AIC values of RCS with different knots. RCS, restricted cubic splines; AIC, Akaike information criterion.

| **RCS Point 1 percentile** | **RCS Point 2 percentile** | **RCS Point 1** | **RCS Point 2** | **ROC Point** | **RCS AIC** |
| --- | --- | --- | --- | --- | --- |
| 0 | 0.35 | 0 | 0.8825 | 1.455 | 147.7785 |
| 0 | 0.4 | 0 | 0.96 | 1.455 | 147.8158 |
| 0 | 0.45 | 0 | 1.0825 | 1.455 | 147.8972 |
| 0 | 0.5 | 0 | 1.17 | 1.455 | 147.9732 |
| 0 | 0.55 | 0 | 1.2225 | 1.455 | 148.0274 |
| 0 | 0.6 | 0 | 1.31 | 1.455 | 148.1164 |
| 0 | 0.65 | 0 | 1.3775 | 1.455 | 148.1775 |
| 0 | 0.7 | 0 | 1.455 | 1.525 | 148.2692 |
| 0 | 0.75 | 0 | 1.455 | 1.8525 | 148.3697 |
| 0 | 0.8 | 0 | 1.455 | 1.95 | 148.3812 |
| 0 | 0.85 | 0 | 1.455 | 2.1175 | 148.391 |
| 0 | 0.9 | 0 | 1.455 | 3.215 | 148.4889 |
| 0 | 0.95 | 0 | 1.455 | 6.16 | 148.5961 |
| 0.05 | 0.35 | 0.41 | 0.8825 | 1.455 | 147.8311 |
| 0.05 | 0.4 | 0.41 | 0.96 | 1.455 | 147.8688 |
| 0.05 | 0.45 | 0.41 | 1.0825 | 1.455 | 147.9534 |
| 0.05 | 0.5 | 0.41 | 1.17 | 1.455 | 148.0322 |
| 0.05 | 0.55 | 0.41 | 1.2225 | 1.455 | 148.088 |
| 0.05 | 0.6 | 0.41 | 1.31 | 1.455 | 148.1782 |
| 0.05 | 0.65 | 0.41 | 1.3775 | 1.455 | 148.2388 |
| 0.05 | 0.7 | 0.41 | 1.455 | 1.525 | 148.3269 |
| 0.05 | 0.75 | 0.41 | 1.455 | 1.8525 | 148.4184 |
| 0.05 | 0.8 | 0.41 | 1.455 | 1.95 | 148.4277 |
| 0.05 | 0.85 | 0.41 | 1.455 | 2.1175 | 148.4346 |
| 0.05 | 0.9 | 0.41 | 1.455 | 3.215 | 148.5218 |
| 0.05 | 0.95 | 0.41 | 1.455 | 6.16 | 148.6203 |
| 0.1 | 0.35 | 0.505 | 0.8825 | 1.455 | 147.8627 |
| 0.1 | 0.4 | 0.505 | 0.96 | 1.455 | 147.9001 |
| 0.1 | 0.45 | 0.505 | 1.0825 | 1.455 | 147.9861 |
| 0.1 | 0.5 | 0.505 | 1.17 | 1.455 | 148.0662 |
| 0.1 | 0.55 | 0.505 | 1.2225 | 1.455 | 148.1228 |
| 0.1 | 0.6 | 0.505 | 1.31 | 1.455 | 148.2133 |
| 0.1 | 0.65 | 0.505 | 1.3775 | 1.455 | 148.2733 |
| 0.1 | 0.7 | 0.505 | 1.455 | 1.525 | 148.3587 |
| 0.1 | 0.75 | 0.505 | 1.455 | 1.8525 | 148.4445 |
| 0.1 | 0.8 | 0.505 | 1.455 | 1.95 | 148.4527 |
| 0.1 | 0.85 | 0.505 | 1.455 | 2.1175 | 148.4578 |
| 0.1 | 0.9 | 0.505 | 1.455 | 3.215 | 148.5389 |
| 0.1 | 0.95 | 0.505 | 1.455 | 6.16 | 148.6327 |
| 0.15 | 0.35 | 0.6 | 0.8825 | 1.455 | 147.896 |
| 0.15 | 0.4 | 0.6 | 0.96 | 1.455 | 147.9338 |
| 0.15 | 0.45 | 0.6 | 1.0825 | 1.455 | 148.0227 |
| 0.15 | 0.5 | 0.6 | 1.17 | 1.455 | 148.1049 |
| 0.15 | 0.55 | 0.6 | 1.2225 | 1.455 | 148.1626 |
| 0.15 | 0.6 | 0.6 | 1.31 | 1.455 | 148.2536 |
| 0.15 | 0.65 | 0.6 | 1.3775 | 1.455 | 148.3129 |
| 0.15 | 0.7 | 0.6 | 1.455 | 1.525 | 148.3949 |
| 0.15 | 0.75 | 0.6 | 1.455 | 1.8525 | 148.4739 |
| 0.15 | 0.8 | 0.6 | 1.455 | 1.95 | 148.4806 |
| 0.15 | 0.85 | 0.6 | 1.455 | 2.1175 | 148.4837 |
| 0.15 | 0.9 | 0.6 | 1.455 | 3.215 | 148.5579 |
| 0.15 | 0.95 | 0.6 | 1.455 | 6.16 | 148.6463 |
| 0.2 | 0.35 | 0.68 | 0.8825 | 1.455 | 147.9195 |
| 0.2 | 0.4 | 0.68 | 0.96 | 1.455 | 147.9603 |
| 0.2 | 0.45 | 0.68 | 1.0825 | 1.455 | 148.0547 |
| 0.2 | 0.5 | 0.68 | 1.17 | 1.455 | 148.1401 |
| 0.2 | 0.55 | 0.68 | 1.2225 | 1.455 | 148.1993 |
| 0.2 | 0.6 | 0.68 | 1.31 | 1.455 | 148.2911 |
| 0.2 | 0.65 | 0.68 | 1.3775 | 1.455 | 148.3497 |
| 0.2 | 0.7 | 0.68 | 1.455 | 1.525 | 148.4283 |
| 0.2 | 0.75 | 0.68 | 1.455 | 1.8525 | 148.5006 |
| 0.2 | 0.8 | 0.68 | 1.455 | 1.95 | 148.5059 |
| 0.2 | 0.85 | 0.68 | 1.455 | 2.1175 | 148.5071 |
| 0.2 | 0.9 | 0.68 | 1.455 | 3.215 | 148.5749 |
| 0.2 | 0.95 | 0.68 | 1.455 | 6.16 | 148.6585 |
| 0.25 | 0.35 | 0.7575 | 0.8825 | 1.455 | 147.9375 |
| 0.25 | 0.4 | 0.7575 | 0.96 | 1.455 | 147.986 |
| 0.25 | 0.45 | 0.7575 | 1.0825 | 1.455 | 148.0894 |
| 0.25 | 0.5 | 0.7575 | 1.17 | 1.455 | 148.1789 |
| 0.25 | 0.55 | 0.7575 | 1.2225 | 1.455 | 148.2399 |
| 0.25 | 0.6 | 0.7575 | 1.31 | 1.455 | 148.3322 |
| 0.25 | 0.65 | 0.7575 | 1.3775 | 1.455 | 148.3898 |
| 0.25 | 0.7 | 0.7575 | 1.455 | 1.525 | 148.464 |
| 0.25 | 0.75 | 0.7575 | 1.455 | 1.8525 | 148.5285 |
| 0.25 | 0.85 | 0.7575 | 1.455 | 2.1175 | 148.5313 |
| 0.25 | 0.8 | 0.7575 | 1.455 | 1.95 | 148.5322 |
| 0.25 | 0.9 | 0.7575 | 1.455 | 3.215 | 148.5922 |
| 0.25 | 0.95 | 0.7575 | 1.455 | 6.16 | 148.6708 |

**Table S8.** Univariate and multivariate logistic analyses of EMZL in patients of derivation cohort. EMZL, extranodal marginal zone B-cell lymphoma; OR, odd ratio; CI, confidence interval; ref, reference.

|  | **EMZL** | | | |
| --- | --- | --- | --- | --- |
|  | **Univariate** | | **Multivariate** | |
| **Variables** | **OR (95%CI)** | ***P*** | **OR (95%CI)** | ***P*** |
| Ordinal IgM |  |  |  |  |
| <0.88 μ/L | ref. | ref. | ref. | ref. |
| 0.88 - 1.46 μ/L | 2.759 (0.91 - 8.39) | **0.074** | 4.262 (1.29 - 14.09) | **0.017** |
| ≥1.46 μ/L | 32 (9.32 - 109.82) | **<0.001** | 37.535 (9.84 - 143.22) | **<0.001** |
| Age <47 | 0.151 (0.04 - 0.53) | **0.003** | 0.141 (0.03 - 0.6) | **0.008** |
| Lacrimal gland involvement | 0.492 (0.19 - 1.26) | 0.14 | 0.34 (0.10 - 1.16) | 0.085 |
| Orbital involvement | 1.79 (0.77 - 4.15) | 0.176 |  |  |
